# Different Effects of Implanting Sensory Nerve or Blood Vessel on the Vascularization, Neurotization, and Osteogenesis of Tissue-Engineered Bone In Vivo

**DOI:** 10.1155/2014/412570

**Published:** 2014-06-30

**Authors:** Jun-jun Fan, Tian-wang Mu, Jun-jun Qin, Long Bi, Guo-xian Pei

**Affiliations:** ^1^Department of Orthopaedics, Xijing Hospital, Fourth Military Medical University, Xi'an 710032, China; ^2^Department of Orthopaedics, Longgang District Central Hospital of Shenzhen, Shenzhen 518116, China; ^3^Department of Orthopaedics, People's Hospital of Guangxi Zhuang Autonomous Region, Nanning 530021, China

## Abstract

To compare the different effects of implanting sensory nerve tracts or blood vessel on the osteogenesis, vascularization, and neurotization of the tissue-engineered bone in vivo, we constructed the tissue engineered bone and implanted the sensory nerve tracts (group SN), blood vessel (group VB), or nothing (group Blank) to the side channel of the bone graft to repair the femur defect in the rabbit. Better osteogenesis was observed in groups SN and VB than in group Blank, and no significant difference was found between groups SN and VB at 4, 8, and 12 weeks postoperatively. The neuropeptides expression and the number of new blood vessels in the bone tissues were increased at 8 weeks and then decreased at 12 weeks in all groups and were highest in group VB and lowest in group Blank at all three time points. We conclude that implanting either blood vessel or sensory nerve tract into the tissue-engineered bone can significantly enhance both the vascularization and neurotization simultaneously to get a better osteogenesis effect than TEB alone, and the method of implanting blood vessel has a little better effect of vascularization and neurotization but almost the same osteogenesis effect as implanting sensory nerve.

## 1. Introduction

The large bone defect caused by the trauma, infection, tumor, or other reasons is a big challenge for surgeons. Autologous bone grafting is considered as the golden standard for the clinical treatment of bone defects but limited by the factors of new wound to the donor site, limited source, and risk of infection [1–5]. Over the last few decades, bone tissue engineering gives us the hope to solve this problem. But bone tissue is a highly vascularized and neurotized tissue [6, 7]. The osteogenesis is influenced by the process of vascularization and neurotization, and these limit the wide clinical application of tissue-engineered bone. Blood vessels have been used to enhance the vascularization and sensory nerve tracts also have been used to enhance the neurotization of tissue-engineered bone to get a better reparative effect in bone defect [8–15]. Constructing a highly vascularized and neurotized tissue-engineered bone according to the theory of biomimetics is a feasible method. But the mechanism of implanting blood vessel or sensory nerve tracts to get a better osteogenesis effect is still unclear. When implanting blood vessel to enhance the vascularization of tissue-engineered bone, there are many nerve fibers distributed in the blood vessel wall and we do not know whether these nerve fibers will enhance the neurotization of tissue-engineered bone or not? By the same token, when implanting sensory nerve tracts to enhance the neurotization of tissue-engineered bone, there are also many capillary networks distributed in the nerve fibers and we do not know whether these capillaries will enhance the vascularization of tissue-engineered bone or not? Which one of these two methods has the better osteogenesis effect in vivo?

Consequently, in our study, sensory nerves tract or blood vessel was implanted into the *β*-TCP/cells constructs, respectively, and used to repair large bone defects in an animal model of rabbit. Our aim was to compare the different effects of implanting sensory nerve tracts or blood vessel on the osteogenesis, vascularization, and neurotization of the tissue-engineered bone in vivo.

## 2. Materials and Methods

### 2.1. Preparation of Tissue-Engineered Bone

Animal experiment was approved by Institutional Animal Care and Use Committee of Fourth Military Medical University. The preparation of tissue-engineered bone was performed as our previous standard procedures described in our published paper [13]. 60 New Zealand male rabbits weighing between 2 and 2.5 kg were purchased from the experimental center of the Xijing Hospital and MSCs were isolated from bone marrow of rabbits. Needle number 16 was used to aspirate the bilateral iliac marrow and the bone marrow sample was anticoagulated by heparin and diluted by Dulbecco's Modified Eagle's Medium (DMEM, Gibco, USA) to 2 mL. Then the lymphocyte separation medium was added with the ratio of 1.5 : 1. The middle single mononuclear cell layer was collected and rinsed by phosphate buffer solution (PBS) for 3 times to count by blood counting chamber after being centrifuged for 30 minutes with 1500 rounds per minute. Then the cells were plated on dish and cultured in DMEM supplemented with 10% fetal bovine serum (FBS, HyClone, USA), 100 U/mL penicillin, and 100 mg/mL streptomycin at 37°C in 5% CO_2_ atmosphere. After 72 h, nonadherent cells were removed. When reaching 70–80% confluence, adherent cells were trypsinized, harvested, and subcultured in osteogenic medium consisting of DMEM supplemented with 15% FBS, 100 nM dexamethasone, 10 mM *β*-glycerophosphate, and 50 mg/L Vitamin C. After 3 weeks of culture, the osteogenic differentiation of MSCs (passage 3) was used to be seeded into the scaffold materials.

**Figure 1:**
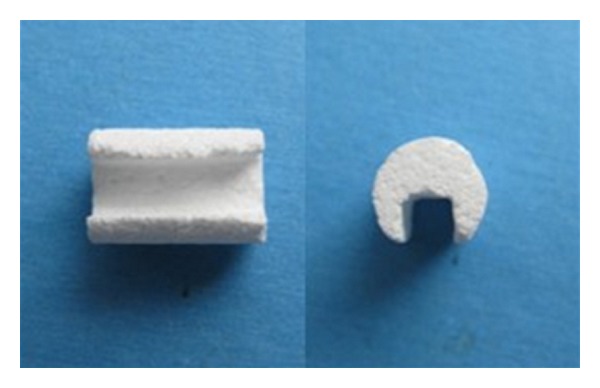
Gross view of *β*-TCP scaffold. *β*-TCP is 15 mm in height and 8 mm in diameter.

The porous *β*-tricalcium phosphate scaffolds (*β*-TCP, 8 mm × 15 mm, pore size 200 *μ*m–300 *μ*m, porosity >85%, Shanghai Bio-lu Biomaterials Company) with lateral groove were prepared and 5 × 10^6^ cells were carefully seeded into each scaffold (Figure 1). The cell-scaffold complexes were put into dishes and moved into incubator for 2 hours so that cells could adhere to the scaffolds. Then, 2 mL medium was carefully added around the complexes. Twelve hours later, an additional 5 mL medium was added and the complexes were incubated in vitro for 3 days prior to implantation.

### 2.2. Animals and Surgical Procedures

**Figure 2:**
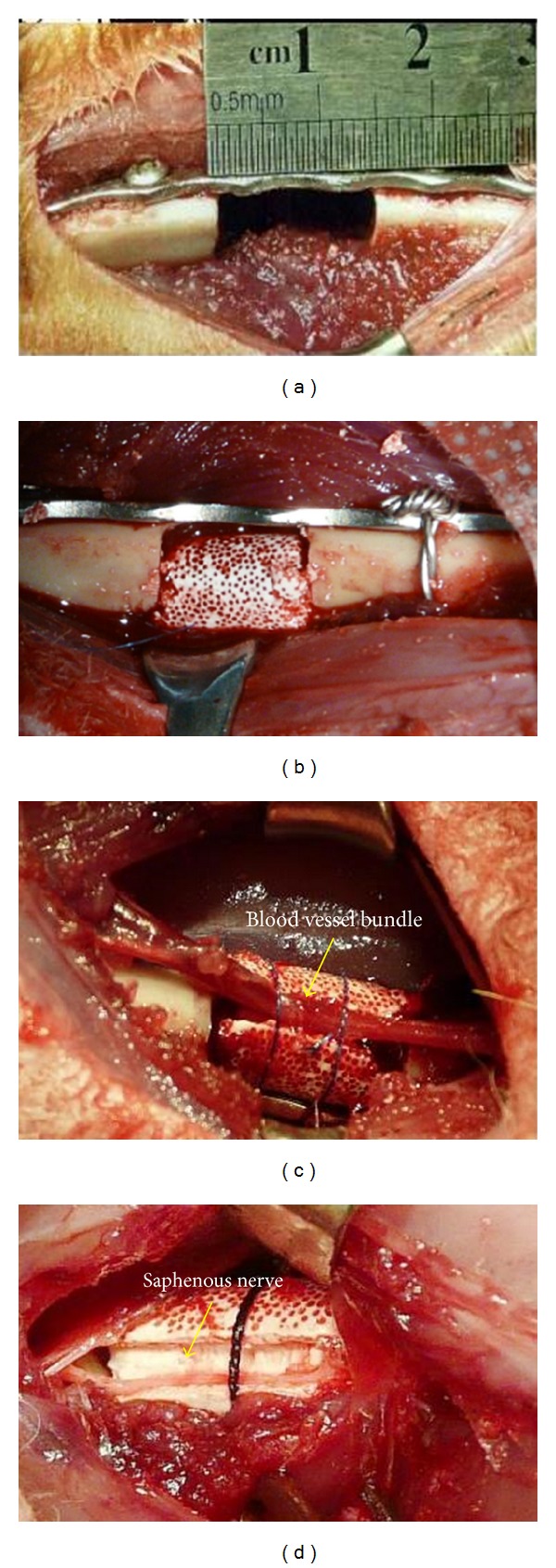
Surgery process of the rabbit model. (a) A 1.5 cm length of femur was intercepted between the second and the third holes of the plate; (b) tissue-engineered bone was imbedded into the defect (group Blank); (c) implanting femoral vascular bundle (group VB); (d) implanting saphenous nerve (group SN).

60 rabbits were randomly divided into three groups with 20 rabbits in each group. For all rabbits, a longitudinal incision was made at the anterolateral femur of the hind limb to expose the femur under general anesthesia. Then a 4-hole steel plate of reconstruction was placed in front of the femur and a femur osteotomy of 1.5 cm length was performed between the second and the third holes of the plate (Figure 2(a)). Tissue-engineered bone was imbedded into the defect site (Figure 2(b)). A second small longitudinal incision was made to expose the saphenous nerve tract and femoral blood vessel including the femoral vein and artery at the femoral triangle. A portion of femoral vein and artery was isolated and then implanted to the side groove of tissue-engineered bone and fixed with sutures (group VB, *n* = 20) (Figure 2(c)). The saphenous nerve tract was regarded as sensory nerve. An appropriate length of saphenous nerve was isolated and implanted into the side groove of tissue-engineered bone (group SN, *n* = 20) (Figure 2(d)). Other animals only had the tissue-engineered bone without the femoral blood vessel or the saphenous nerve (group Blank, *n* = 20). All incisions were closed using nonabsorbable sutures, and 400,000 U of penicillin was administrated daily by intramuscular injection for three days.

### 2.3. Histological Analysis

Five animals of each group were collected randomly and sacrificed to collect the bone graft at 4, 8, and, 12 weeks. Specimens were fixed in 4% buffered paraformaldehyde, decalcified in 50 mM ethylene diaminetetraacetic acid (EDTA), serially dehydrated, infiltrated in isoamyl alcohol, embedded in paraffin, and sectioned into 4 *μ*m thickness. 20 sections were made for each sample with 5 being for staining with haematoxylin and eosin. The degree of bone regeneration was calculated as percent area of new bone within the bone graft and measured using the image analysis software (Image-Pro Plus 6.0, USA). All measurements were performed within a region of interest with the diameter of 5 mm in each section. The mean percentage of new bone area was calculated from all sections of each sample and compared between groups.

### 2.4. Immunohistochemistry Analysis

The other fifteen sections of each sample were randomly divided into three groups and used for immunostaining. Incubation of the sections with 3% H_2_O_2_ for 10 min blocked the nonspecific binding. The antigen was repaired by heat. The sections were incubated with 10% normal goat serum in PBS for 20 minutes to increase the penetration of the antibodies. The sections were then incubated in one of the primary antibodies separately (anti-CD34 1 : 100, anti-CGRP 1 : 4000, and anti-NPY 1 : 4000, Wuhan Boster Biological Technology Ltd., China) for 2 h and incubated in second antibodies for 20 min at room temperature. Then they were further processed using SABC immunostaining kit (Wuhan Boster Biological Technology Ltd.) according to the manufacturer's instruction. Known CD34, CGRP, and NPY expression slides were used as positive controls and PBS was replaced with primary antibodies as negative control.

The number of new blood vessels at the implantation site was determined by analyzing the sections immunostained with anti-CD34 antibodies and the total number of vessels on complete implant sections was recorded with Image-Pro Plus software. A vascular section was defined as a vessel with a recognizable lumen. Any single endothelial cell or cluster of endothelial cells clearly separated from adjacent vessels was considered as one countable vessel. The evaluation was performed by three individuals who were blinded to the treatments. Then, their counts were averaged as the blood vessel count of each sample.

### 2.5. Gene Expression Analysis

About 40 mg new bone tissues were collected from different parts of the tissue engineering bone graft before the histological staining. In order to analyze messenger ribonucleic acid (mRNA) expression, tissue was grinded and homogenized in 1 mL of TRIzol Reagent (Invitrogen, Carlsbad, CA, USA), and total RNA was extracted from tissue samples according to the manufacturer's instructions and quantified using spectrophotometric analysis. RNA quantity was assessed using gel electrophoresis. Reverse transcription (RT) was performed with the SuperScript First-Strand Synthesis System for RT-PCR (Invitrogen, Grand Island, NY, USA) according to the manufacturer's protocol. cDNA was used as a template, and quantitative real-time RT-PCR was performed with Bio-rad 170-9780 iQ5 Multicolor Real-Time PCR Detection System (BioRad, USA) according to the manufacturer's protocol. Primer pairs were as follows: 5′-AGCAGGAAGACCAGGAGCAG-3′ (forward) and 5′-CACATTGGTGGGCACAAAGT-3′ (reverse) for CGRP (Invitrogen), 5′-GCGACACTACATCAATCTCATCA-3′ (forward) and 5′-GAAGGGTCTTCAAGCCTGGT-3′ (reverse) for NPY (Invitrogen), and 5′-CCGTCTTCCCCTCCATCGT-3′ (forward) and 5′-TTCGTGCTCGATGGGGTACT-3′ (reverse) for *β*-actin (Invitrogen). The PCR mixture consisted of 10x buffer, 25 mM MgCl_2_, 25 mM dNTPs (TaKaRa, Japanese), ultrapure water, 10 mM upstream primer, 10 mM downstream primer, 50 × sybr, 5 u/*μ*L Taq enzyme, and template in a final volume of 25 *μ*L. Values were expressed as relative percentages compared to *β*-actin mRNA levels.

### 2.6. Ink Perfusion for Angiogenesis Observation

At 12 weeks, five animals in each group were selected to observe the angiogenesis by perfusion of prepared Chinese ink into blood vessels. After general anesthetization, an incision was made at the groin area to expose femoral artery and vein. Firstly, the femoral artery was blocked and a catheter was inserted into the artery. A solution of 0.4% heparin was perfused through the catheter. At the same time the femoral vein was cut to observe the color of backflow fluid until the fluid was clear. Then Chinese ink was perfused though the catheter. When the backflow fluid reached the femoral vein and the skin of the animal became black, the femoral vein was ligated. After 5 min of ink perfusion, all the blood vessels around femur were ligated and the bone graft was harvested for histological examination. After that, the specimen was fixed in 4% buffered paraformaldehyde and decalcified in 50 mM ethylenediaminetetraacetic acid (EDTA). Sections with 10 *μ*m thickness were prepared. Five sections of each sample were collected to observe the situation of angiogenesis. The number of new blood vessels was determined by analyzing the sections immunostained with ink and the total number of vessels on complete implant sections was recorded with Image-Pro Plus software. A vascular section was defined as a vessel with a recognizable lumen. The evaluation was performed by three individuals who were blinded to the treatments. Then, their counts were averaged as the blood vessel count of each sample.

### 2.7. Statistics

All data were analyzed using SPSS software. The data were expressed as mean ± standard deviation (SD) and levels in all groups were compared by a one-way analysis of variance and Student's *t*-test; *P* values less than 0.05 were considered significant.

## 3. Results

### 3.1. Histological Assessment

**Figure 3:**
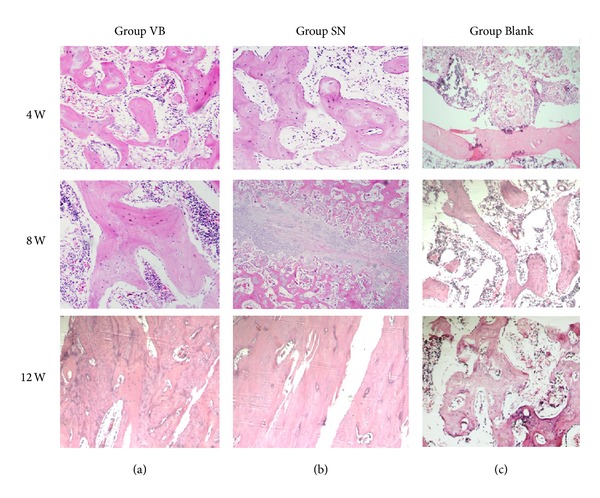
Examples of H&E staining. The HE staining showed that the formations of new bone in group VB (a) and group SN (b) were better than that in group Blank (c) and there was no obvious difference between VB and SN at 4, 8, and 12 weeks (HEX100).

**Figure 4:**
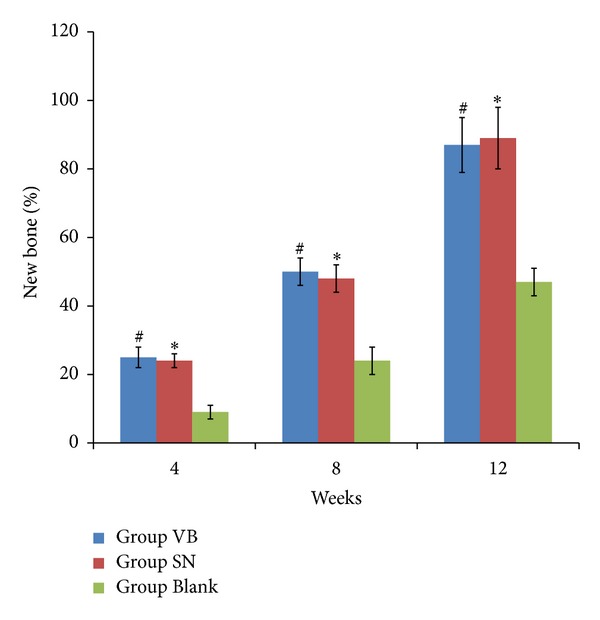
The percentage of new bone formation in all groups at 4, 8, and 12 weeks postoperatively. The percentage of new bone formation in all groups was gradually increased over time and lower in group Blank than in the other two groups at different times, and there were no significant differences between the group SN and group VB at 4, 8, and 12 weeks after surgery. *P < 0.05 versus group Blank; ^#^ *P* > 0.05 versus group SN.

H&E staining showed that no inflammatory response was observed and the new bone tissue was gradually increased in all groups over time with the degradation of the scaffolds. At 12 weeks, newly formed bone was showed extensively and most of the scaffolds had been degraded in groups VB and SN. No significant difference was found between group SN and group VB. Similar histological changes occurred in group Blank, but the new bone formation was less and slower than the other two groups (Figure 3). The formation of new bone was gradually increased in all groups over time, and the amount of new formed bone was higher in group SN and group VB than in group Blank at each time point (*P* < 0.05). No significant difference was found between group SN and group VB at each time point (*P* > 0.05) (Figure 4).

### 3.2. Immunohistochemistry Assessment

**Figure 5:**
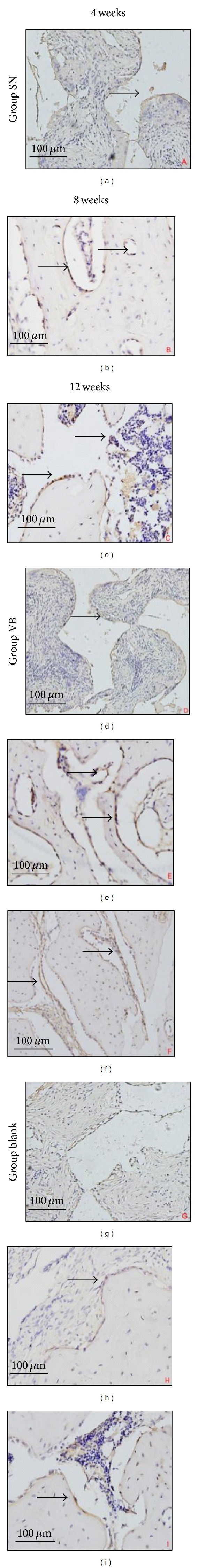
Staining of CGRP in three groups at 4, 8, and 12 weeks after surgery (black arrows). CGRP staining showed that there was more intensive staining in groups SN and VB than in group Blank at different times and was mainly around the periphery of newly generated bone tissues (×400, scale bar represents 100 *μ*m).

**Figure 6:**
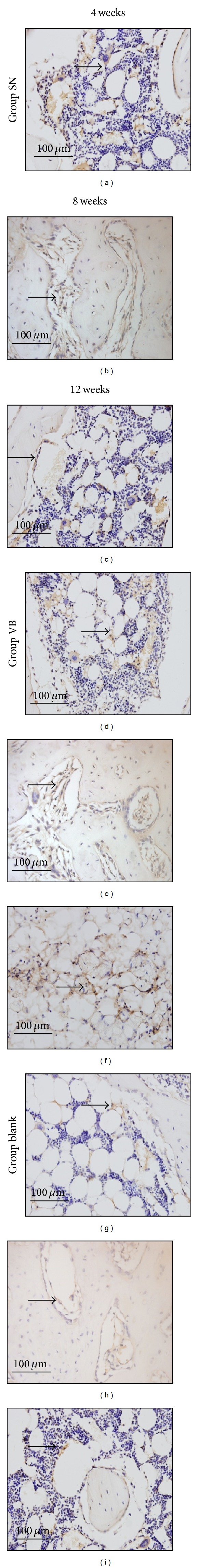
Staining of NPY at 4, 8, and 12 weeks after implantation. NPY staining showed that there was more intensive NPY in groups SN and VB than in group Blank at different times, and the NPY staining was mainly localized to the periphery of the newly generated bone tissues and the blood vessels (black arrows) (×400, scale bar represents 100 *μ*m).

The positive staining for CGRP or NPY was found in newly regenerated tissue in group VB and group SN at week 4 postoperatively with more intensive positive staining in group VB than group SN. The intensity of positive CGRP or NPY staining increased with time and peaked at week 8 in all groups and remained less staining in group SN compared to group VB. Group Blank had the least intensity of positive staining of both CGRP and NPY. In addition, it was found that the locations of CGRP and NPY staining were mainly around the blood vessels and the newly formed bone tissues. At week 12 postoperatively, there was a little decrease of positive CGRP and NPY staining in all groups. However, the positive staining of CGRP or NPY in group SN remained less intensive than that of group VB and still least intensive in group Blank (Figures 5 and 6).

### 3.3. Quantification of Vascular Regeneration

**Figure 7:**
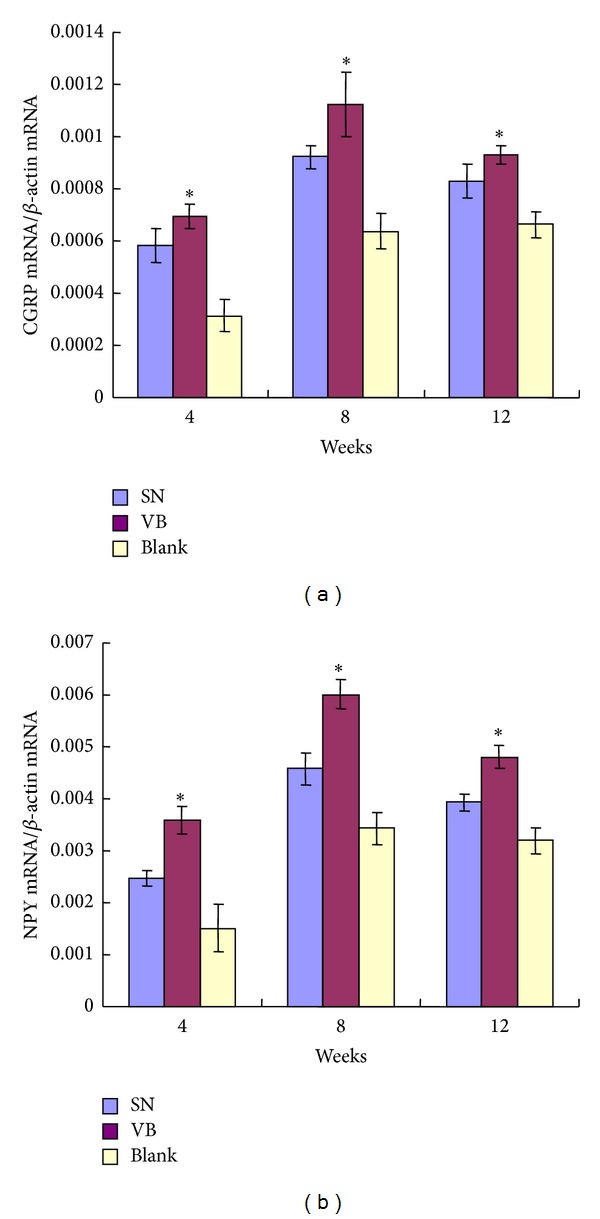
Comparison of the CGRP and NPY mRNA at different time points after implantation. Transcript levels by real-time PCR for CGRP mRNA (a) and NPY mRNA (b) in the new bone tissues were shown as a fold ratio relative to the expression of the *β*-actin mRNA at 4, 8, and 12 weeks after surgery, respectively. The mRNA levels in groups VB and SN were higher than in group Blank and were highest in group VB at all three time points. *P < 0.05.

The number of new blood vessels determined by the positive staining of CD34 in each group was increased at 8 weeks and then decreased at 12 weeks. The number of new blood vessels in groups VB and SN was both higher than in group Blank but was highest in group VB at all three time points (*P < 0.05) (Figure 7).

### 3.4. CGRP and NPY mRNA Expression

**Figure 8:**
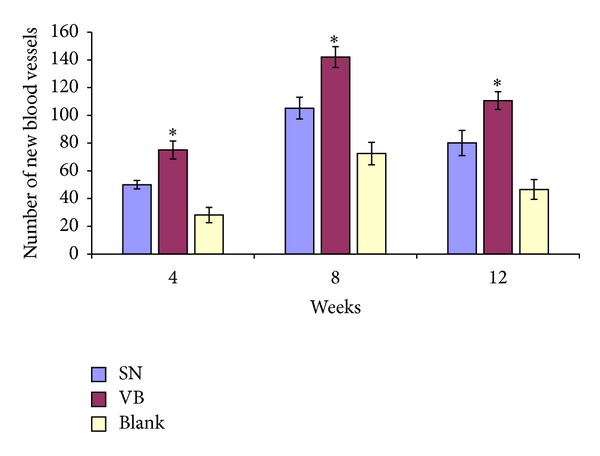
The number of new blood vessels at different time points after implantation. The number of new blood vessels counted by the expression of CD34 in all three groups was highest at 8 weeks and lowest at 4 weeks. The number of new blood vessels in groups VB and SN was higher than in group Blank and was highest in group VB at all three time points. *P < 0.05.

The transcript expression of CGRP or NPY was quantified by real-time RT-PCR and mRNA levels in the new bone tissue of all groups were increased at 8 weeks and then decreased at 12 weeks, but the level was still higher than that of 4 weeks. The mRNA levels of CGRP and NPY in group VB and group SN were significantly higher than that of group Blank and were highest in group VB at all the three time points (*P < 0.05) (Figure 8).

### 3.5. Ink Perfusion of New Blood Vessels

**Figure 9:**
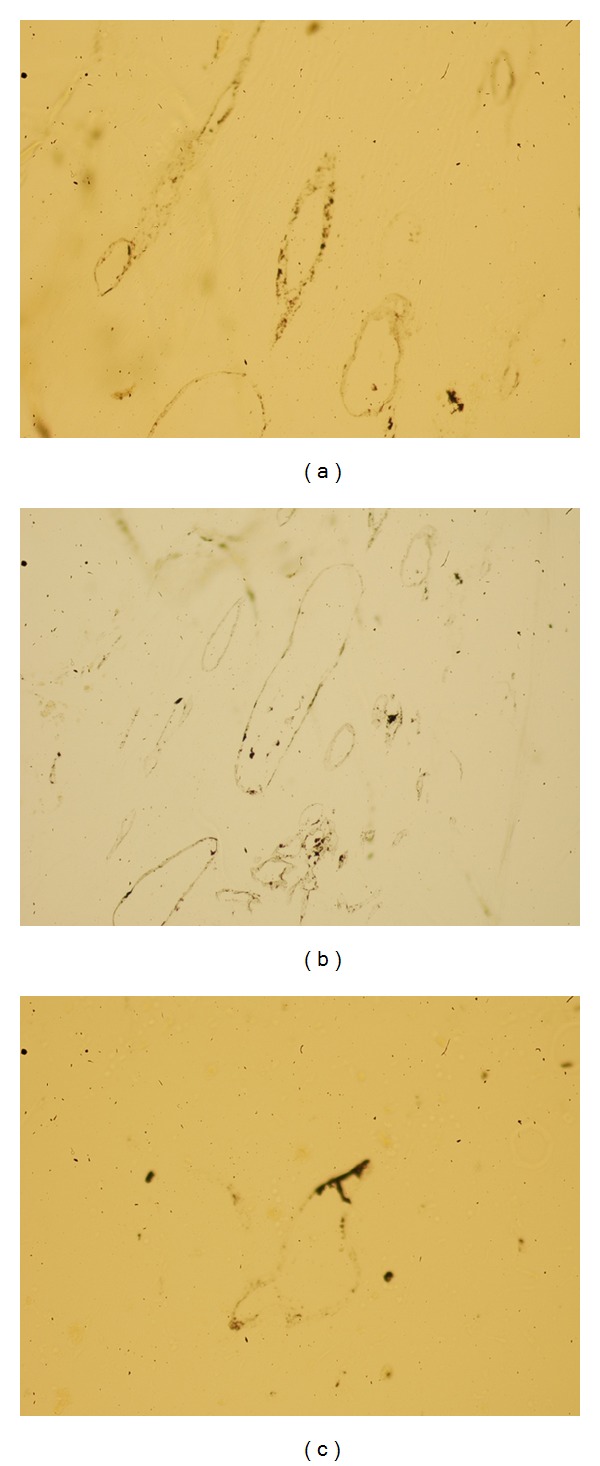
Samples of Chinese ink perfusion with HE staining at 12 weeks. (a) Group SN; (b) group VB; (c) group Blank; ×200. At 12 weeks after implantation, the ink staining of new blood vessels was more intensive in groups SN and VB than in group Blank and was most intensive in group VB; the vascular morphology was better and more mature in groups SN and VB than in group Blank and was best in group VB.

At 12 weeks after implantation, the ink staining of new blood vessels was more intensive in groups SN and VB than in group Blank and was most intensive in group VB; the vascular morphology was better and more mature in groups SN and VB than in group Blank and was best in group VB (Figure 9).

## 4. Discussion

Bone tissue is a highly vascularized and neurotized tissue. The osteogenesis is influenced by the process of vascularization and neurotization, and this limits the wide clinical application of tissue-engineered bone. Constructing a highly vascularized and neurotized tissue-engineered bone according to the theory of biomimetics has become a useful method for repairing the large bone defect. Over the last few years, many researchers have used blood vessel to enhance the vascularization and sensory nerve tracts to enhance the neurotization of tissue-engineered bone to get a better reparative effect in bone defect [8–14]. But the mechanism of implanting blood vessel or sensory nerve tracts to get a better osteogenesis effect is still unclear. There are many nerve fibers distributed in the blood vessel wall and there are also many capillary networks distributed in the nerve fibers. The relationship of vascularization and neurotization in the tissue-engineered bone still needs to be discovered.

The intrinsic vascularization of tissue-engineered bone can be induced via a blood vessel located centrally in the bone graft by microsurgical technique. There are many reports about the usage of the blood vessel in biomaterial results in splendid osteogenesis and vascularization of the bone grafts [8, 9, 16, 17]. But when implanting blood vessel to enhance the vascularization of tissue-engineered bone there are many nerve fibers distributed in the vascular bundles wall and we do not know whether these nerve fibers will enhance the neurotization of tissue-engineered bone or not? In our study, we found that the mean number of new blood vessels in the new bone determined by immunohistochemistry of CD34 was highest in group VB. And, at 12 weeks, the result of vascular ink dyeing showed that the quantity and quality of newborn vascular network were also best in group VB. The results of CD34 and ink dyeing were consistent with previous literature and showed that the implanting blood vessel could enhance the vascularization of tissue-engineered bone. In the meanwhile, the CGRP and NPY expression determined by immunohistochemistry and RT-PCR was also highest in group VB at all the time points in our study. This result showed that implanting blood vessel could also enhance the neurotization of tissue-engineered bone besides the vascularization. The reason of enhancing the neurotization by implanting the blood vessel may be related with the nerve fibers distributed in the blood vessel wall. But the actual reason still needs to be studied in the further research.

Bone tissue is not only a highly vascularized tissue but also a highly neurotized tissue. By the anatomy study of the bone tissue, many researches have proved that there were dense and intimate networks of nerve in the bone tissue and the nerve networks had contact with bone cells [18–21]. Then many researchers found the intimate relationship between the bone formation and the nerve networks development and proved that nerve networks played a very important role in the process of bone formation [22–24]. These neuropeptides including calcitonin gene-related peptide (CGRP) and nerve peptide Y (NPY) enhanced the proliferation of osteoblasts in vitro and inhibited the bone resorbing activity of isolated osteoclasts by regulating different cytokines pathways [25–32]. The ability of peptidergic nerve fiber to regulate the bone formation can be used to construct the neurotized tissue-engineered bone by embedding the sensory nerve fiber into the center of bone graft [12–14]. In our study, we found that the CGRP and NPY expression determined by immunohistochemistry and RT-PCR was higher in group SN than group Blank at all the time points. This result showed that implanting sensory nerve bundles could enhance the neurotization of tissue-engineered bone. In the meanwhile, larger number of new blood vessels in the new bone determined by immunohistochemistry of CD34 was also found in group SN compared with group Blank at all the time points. And, at 12 weeks, the result of vascular ink dyeing also showed the quantity and quality of newborn vascular network were better in group SN than in group Blank. The results of CD34 and ink dyeing showed that implanting sensory nerve fiber could also enhance the vascularization of tissue-engineered bone besides the neurotization. The reason of enhancing the vascularization by implanting the sensory nerve fiber may be related with the capillary network distributed in the sensory nerve fiber. These neuropeptides of CGRP and NPY secreted from the sensory nerve are also associated with the dilation and the constriction of blood vessels through interaction with neuropeptide receptors present on both endothelial cells and vascular smooth muscle cells via endothelium-dependent or endothelium-independent mechanisms, depending on the vessel type and species [33–38].

In our study, the group of implanting blood vessels had higher expressions of CD34, CGRP, and NPY than those of implanting sensory never and blank groups. The result of vascular ink dyeing also showed the quantity and quality of newborn vascular network were better in the group of implanting blood vessels at 12 weeks. But the osteogenesis observed by histology showed no significant difference between the groups SN and VB at 4, 8, and 12 weeks postoperatively. This result is out of our expectation. The reason is still unclear and we think that the osteogenesis of tissue-engineered bone may be influenced by other factors besides the vascularization and neurotization. Whatever, we found that implanting either blood vessels or sensory nerve into the tissue-engineered bone could significantly enhance both the vascularization and neurotization simultaneously to get a better osteogenesis, and the method of implanting blood vessel has a little better effect of vascularization and neurotization but almost the same osteogenesis effect as the sensory nerve. So it is feasible to implant vascular bundles into the tissue-engineered bone to construct vascularized and neurotized tissue-engineered bone and to repair large bone defect. And implanting the sensory nerve fibers was also an alternative method to be used in some clinical conditions.

## 5. Conclusion

In conclusion, we compared the different effects of implanting sensory nerve tracts or blood vessel on the osteogenesis, vascularization, and neurotization of the tissue-engineered bone in vivo. We found that implanting either blood vessel or sensory nerves tract into the tissue-engineered bone could significantly enhance both the vascularization and neurotization simultaneously to get a better osteogenesis compared with using the tissue-engineered bone only, and the method of implanting blood vessel has a little better effect of vascularization and neurotization but almost the same osteogenesis effect as implanting the sensory nerve. So it is feasible to implant blood vessel or sensory nerve into the tissue-engineered bone to construct vascularized and neurotized tissue-engineered bone and repair the large bone defect depending on the clinical conditions.
